# Computational Approach for Structural Feature Determination of Grapevine NHX Antiporters

**DOI:** 10.1155/2019/1031839

**Published:** 2019-01-09

**Authors:** Mariem Ayadi, Rayda Ben Ayed, Rim Mzid, Sami Aifa, Mohsen Hanana

**Affiliations:** ^1^Laboratory of Extremophile Plants, Center of Biotechnology of Borj-Cédria, BP 901, Hammam-lif 2050, Tunisia; ^2^Laboratory of Molecular and Cellular Screening Processes, Center of Biotechnology of Sfax, University of Sfax, Sidi Mansour Road, P.O. Box 1177, 3018 Sfax, Tunisia

## Abstract

Plant NHX antiporters are responsible for monovalent cation/H^+^ exchange across cellular membranes and play therefore a critical role for cellular pH regulation, Na^+^ and K^+^ homeostasis, and salt tolerance. Six members of grapevine NHX family (VvNHX1-6) have been structurally characterized. Phylogenetic analysis revealed their organization in two groups: VvNHX1-5 belonging to group I (vacuolar) and VvNHX6 belonging to group II (endosomal). Conserved domain analysis of these VvNHXs indicates the presence of different kinds of domains. Out of these, two domains function as monovalent cation-proton antiporters and one as the aspartate-alanine exchange; the remaining are not yet with defined function. Overall, VvNHXs proteins are typically made of 11-13 putative transmembrane regions at their N-terminus which contain the consensus amiloride-binding domain in the 3^rd^ TM domain and a cation-binding site in between the 5^th^ and 6^th^ TM domain, followed by a hydrophilic C-terminus that is the target of several and diverse regulatory posttranslational modifications. Using a combination of primary structure analysis, secondary structure alignments, and the tertiary structural models, the VvNHXs revealed mainly 18 *α* helices although without *β* sheets. Homology modeling of the 3D structure showed that VvNHX antiporters are similar to the bacterial sodium proton antiporters MjNhaP1 (*Methanocaldococcus jannaschii*) and PaNhaP (*Pyrococcus abyssi*).

## 1. Introduction

The NHX antiporters family mediates cation/proton exchange across different cellular membranes, using electrochemical gradients generated by proton translocating enzymes, namely, H^+^-ATPase in the plasma membrane and vacuolar ATPase and pyrophosphatase within the intracellular compartments [1, 2]. Members of this NHX family are found in all living organisms and cells, from prokaryotic to eukaryotic. In plants, they are thought to play function in several biological roles through their cation/proton exchange activity, such as cellular pH regulation, potassium homeostasis, growth, developmental processes, and osmotic stress tolerance [3–5]. In grapevine* Vitis vinifera* L., one member (VvNHX1) of the VvNHX family has been already characterized and reported by Hanana et al. [5]; the remaining members still to be described and studied. Although the number of* Vitis vinifera* protein sequences is growing considerably since the inception of genome sequencing, however, functional annotation of these proteins is often less accurate or missing. Therefore, functional studies and biological roles identification are clearly needed. Protein structure determination, whether performed through X-ray crystallography and NMR spectroscopy or computational and* in silico* methods, would improve our knowledge about their functions and biological roles. However, protein crystallization, mainly transmembranous, is a staggering task due to the problem of solubility and stability and is therefore time and cost consuming. Computational methods and bioinformatics tools would be an interesting alternative for protein structure prediction. Indeed, several computer tools and programs for structure prediction have been developed and are now available [6]. In this context, our work focuses on structural features determination of grapevine NHX antiporters via the use of computational and* in silico* methods. The objective of our study is to predict the three-dimensional structure of* Vitis vinifera* NHX antiporter protein family through homology modeling and examine its physicochemical properties using* in silico* approaches. Biocomputational analyses of the targeted proteins were performed using an array of online bioinformatics tools and databases. The homology model was developed using different software packages and the best model was selected upon evaluation.

## 2. Material and Methods

### 2.1. Database Search and Identification of Grapevine* VvNHX* Genes

To search for putative NHX genes in the genome of* Vitis vinifera*, a keyword search “sodium/hydrogen antiporter” was performed in National Center for Biotechnology Information (NCBI) database. The selected genes were used for a basic local alignment search tool (BLAST) on the grapevine genome in the Phytozome database V12 (http://www.phytozome.net/). To validate the reliability of these potential candidates, the Pfam database (http://pfam.sanger.ac.uk/search) was exploited to confirm each VvNHX candidate as a true member of NHX family.

### 2.2. Phylogenetic Relationships and Identification of Conserved Protein Motifs

Alignments were performed using the Clustal W module from MEGA6 software [7] which help to draw the unrooted phylogenetic tree [7] using the neighbor-joining method with statistical bootstrapping procedure involving 1000 replicates. MEME software (Multiple Expectation Maximization for Motif Elicitation) (MEME version 4.10.2) (http://meme.sdsc.edu/meme/intro.html) led us to the identification of conserved motifs and the description of their position and frequency [8]. The used parameters in the analysis were repetitions number: 3, maximum number of motifs: 10; minimum motif width: 10; and maximum motif width: 50. Structural motif annotation was used directly at the Conserved Domain Database (CDD) (http://www.ncbi.nlm.nih.gov/cdd/).

### 2.3. Structural Features Characterization

The analyzing tool of protein primary structures is provided by the Protpram ExPASy server (Expert Protein Analysis System; https://web.expasy.org/protparam/) [9] including MW (Molecular Weight), IEP (Isoelectric point), and composition of amino acids. The subcellular localization of VvNHX proteins was predicted with WOLF PSORT [10] (https://wolfpsort.hgc.jp/) and CELLO Prediction (http://cello.life.nctu.edu.tw/) [11]. Sequence similarity was calculated with the global alignment tool using BLOSUM50. Transmembrane (TM) helix prediction was performed by the consensus extracted from ARAMEMNON database which has been planned to collect various computational predictions for plant membrane proteins (Eiconda_v1, TmHMM, TMMOD, MemSAT_v3, Philius, Phobius, DAS-TMfilter, PredTmr-v1, Scampi, SosuiG_V1.1, THUBUP_v1, Hmmtop-V2, TmPred, and TopPred-v2) [12]. In particular, the alignment of these VvNHX proteins was drawn with PRALINE multiple sequence alignment using DSSP [13] and PSIPRED [14] to obtain secondary structure prediction. Also, the secondary structure of the protein was predicted by NPS Secondary Structure Prediction Method [15]. Structural disorder was examined using PONDR-FIT accessible from the platform of DisProt [16] using the VSL2B predictor option [17]. All curves obtained are overlapped by a tool of plot on the R graphic [18]. The 3D models of the targeted VvNHX proteins were constructed using protein structure homology model building programs PHYRE2 [19] (http://www.sbg.bio.ic.ac.uk/phyre2/html/page.cgi?id=index). The FASTA sequences of the query proteins were entered and the intensive mode was selected to attain 3D models. Molecular graphics and analyses were performed with the PyMOL Molecular Graphics System, Version 2.0 Schrödinger, LLC.

Posttranslational modification analyses were predicted using the CBS server (Center for Biological Sequence Analysis) (http://www.cbs.dtu.dk/services). Supposed phosphorylation sites were analyzed using KinasePhos (http://kinasephos.mbc.nctu.edu.tw) [20].

## 3. Results

### 3.1. Members of* VvNHXs *Family

6 members of* VvNHX* genes family were retrieved from the NCBI database in the whole grapevine genome after a procedure of several plant NHX sequences blast request and further screening for the presence of “sodium/hydrogen exchanger family” domain. Therefore, 6 nonredundant genes were found and confirmed as* NHX* genes in the grape genome, and then named* VvNHX1* to* VvNHX6 *(Table 1). The prediction of their subcellular localization showed that most of VvNHXs belong to intracellular membranes.

### 3.2. Phylogenetic Groups and Motif Analysis of VvNHX Proteins

**Figure 1:**
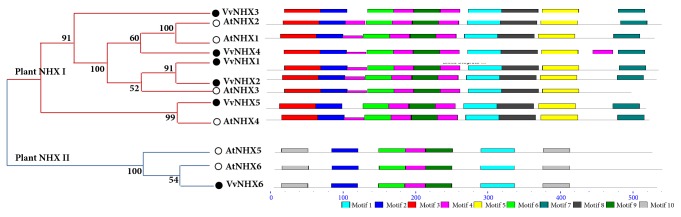
Conserved protein motifs and phylogeny of VvNHXs. Phylogeny and schematic representation of the 10 conserved motifs in NHX proteins of Grapevine and Arabidopsis. The tree was constructed with VvNHX and AtNHX proteins using the neighbor-joining algorithm of MEGA 6 [7]. The bootstrap consensus tree was inferred from 1000 replicates. Scale bar corresponds to 0.1 amino acid substitution per residue. Motif analysis was performed using MEME 4.0 software as described in Methods. Different motifs, numbered 1–10, are displayed in different colored boxes. A detailed motif introduction for NHXs proteins is shown in Table 2.

The phylogenetic tree (Figure 1) drawn using both Grapevine and Arabidopsis NHX protein sequences reveals the existence of two major groups of VvNHX antiporters (I and II) that gather either with AtNHX1–4 (group I) or AtNHX5/6 (group II). VvNHX6, presumed to be of endosomal location, is the only member of group II.

Furthermore, in order to identify conserved motifs and consensus domains constituting the NHX proteins, the online MEME Suite (v4.8.2) program was used (Figure 1). The sequence details of each motif are shown in Table 2. Analogous motifs were shared between the two different NHX groups, suggesting common conserved functions inside the NHX family. The first motif corresponding to aspartate-alanine exchange (Asp-Al_Ex) pattern is found in all groups between TM8 and TM9 regions. The conserved motifs 2 and 4 were characterized as Monovalent Cation: Proton Antiporter-1 (CPA1), which were broadly distributed in all NHX protein sequences. The CPA1 family members allow the electroneutral exchange of monovalent cations for H^+^ in a pH-dependent way [23]. Motifs 6 and 9, present in all VvNHXs, are conserved sequences in sodium/proton exchangers. The motifs 3, 5, 7, and 8 were found only within VvNHXs belonging to group I, while the motif 10 was distributed exclusively in VvNHX6 and in twice, though the function of these motifs are still unknown.

### 3.3. Structural Characterization of VvNHX Proteins

#### 3.3.1. Primary Structure

The size of encoded proteins of the identified* VvNHX* genes ranged from 524 to 541 amino acids (aa), with an average of 535 aa, and the corresponding predicted molecular masses ranged from 58.4 to 60.1 kDa, with an average of 59.4 kDa (Table 1). The computed IEP of these proteins was ~7.13 on average, indicating that they are likely to precipitate in either acidic or basic buffers and can be maintained within a neutral buffer.

The VvNHX1-6 proteins shared 30 to 80% of similarity (Table 3). VvNHX1-5 (group I) in particular shared more than 80% of identity among VvNHX1 and 2 and more than 70% of identity among VvNHX1 and 4. However, VvNHX6 (group II) shares the lowest similarity (30%) with VvNHX3 and 5, although displaying similar lengths. This wide range of variability among VvNHX members would be synonymous of functional diversification.

Main represented aa (Table 4) of these NHX members are leucine (13%), alanine (9%), glycine (9%), valine (9%), and serine (8%). The least common aa residues were cysteine and tryptophan which accounted for ~1% of the protein's primary structure. The low amounts of cysteine residues indicated that the probabilities of disulfide bond formation were low. Leucine, alanine and valine are hydrophobic, aliphatic and nonpolar amino acids and thus expected to be inside the protein or within lipid membranes. Glycine, the smallest aa, known for its flexibility, would allow the protein twist and rotation. Serine, a polar aa, is qualified as hydrogen bond donor. The contribution of most readily oxidable amino acids was only 10% in all VvNHXs, which could be gainful to get better stability of targeted proteins under stress. These assumed that the targeted vacuolar-NHX protein of* Vitis vinifera* is hydrophobic in nature. Negatively and positively charged aa number of VvNHXs ranged from 30 to 45 (Table 4). However, the residual and global charge (calculated at pH 7) of the whole NHX protein varies from negative (VvNHX2, 4, and 5), neutral (VvNHX1), positive (VvNHX3) to highly positive for VvNHX6 (Table 4), depending on the pH environment of the different extra-membrane VvNHX segments.

#### 3.3.2. Secondary Structure

**Figure 2:**
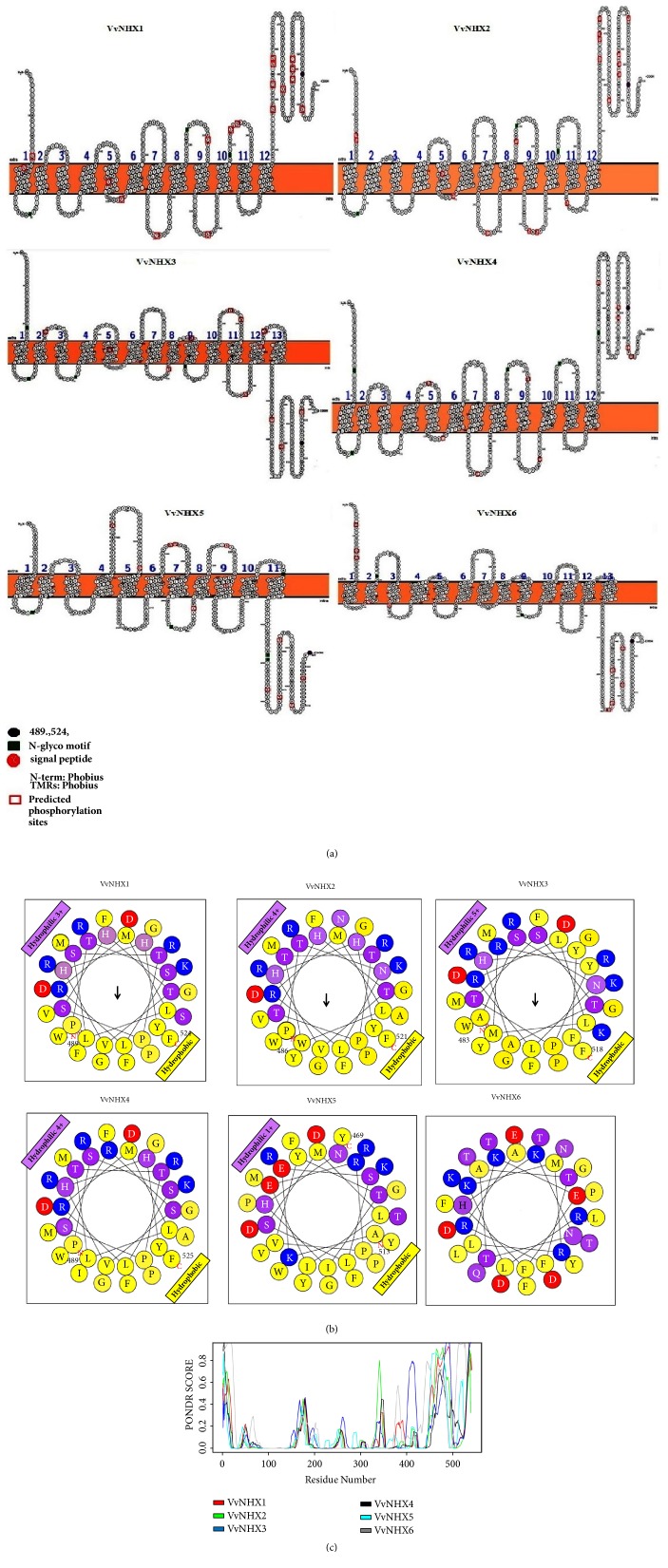
Structural analysis of VvNHX proteins. (a) Transmembrane domains in VvNHX proteins constructed with Protter [21]. (b) Helicoidal representation of calmodulin (CaM) domain in all VvNHXs constructed with Heliquest [22]. (c) VvNHX proteins contain large unstructured regions. The disorder of NHX proteins was predicted using VSL2B from Predictor Of Naturally Disordered Regions (PONDR)[16]. The plot was drawn with R.

Data extracted from ARAMEMNON database [12] indicated that the amino acid sequences of VvNHXs comprise a predicted secretion pathway. Topological prediction analyses of transmembrane (TM) domains of VvNHXs showed a range from 11 to 13 TM domains, 23 residues for each (Figure 2(a); supplementary file 1). Our MEME analysis, set with the mentioned parameters, led to the identification and localization of 10 motifs within the VvNHX proteins (Figure 1). Motifs 2 and 4 are the functional CPA1 conserved domain of NHE proteins and are widely distributed in all VvNHXs proteins. All the members of VvNHXs contained a conserved sequence FFI/LY/FLLPPI in the motif 2, at a conserved position of the 3^rd^ TM domain (Supplementary file 1), corresponding to an amiloride-binding site in mammals that is known to be an inhibitor of eukaryotic NHXs [24]. Harris and Fliegel [25] have demonstrated that sodium ions and amiloride molecule interact at unique regions of NHE-like Na^+^/H^+^ transporters. Cation-binding site was predicted to be located in between the 5^th^ and 6^th^TM domain. The NHX TM domains are well conserved in different sequences; however, variations are seen at N- and C-termini of amino acids sequences (Figure 2(a), supplementary file 1). In contrast, VvNHX1-5 proteins (Group I) have a calmodulin (CaM) binding domain at the end of their C-termini, like the one identified in AtNHX1 [26]. This same domain was identified in VvNHX1 by Hanana et al. [5] from 489 to 524 amino acids. Helical wheel representation (Figure 2(b)) of these peptide regions displayed an amphipathic pattern. However, VvNHX6 does not share a high similarity of this region in its C-terminus, probably because of its endosomal location and its implication in other functions.

The two dimensional secondary structure of NHX protein sequences was predicted using GORIV of NPS [15]. Secondary structure prediction showed that all VvNHXs contain around 30%  *α*-helices, 25% extended strands, and 45% random coils and loops (Table 5). In light of the above, the presence of helices in the protein makes it more flexible for folding, which might increase protein interactions [27]. A secondary factor that influences proteins' stability is the presence of extensive hydrogen bonds.

To further analyze their structural flexibility, we carried out sequence analysis with PONDR-FIT, a computational tool for prediction of structured and unstructured regions [16]. As shown in Figure 2(c), several regions of VvNHXs are disordered. Unexpectedly, this disorder was roughly preserved all along the VvNHXs. These unstructured regions are registered along more than two-thirds of each sequence. Surprisingly, unstructured regions were observed especially in N- and C-termini, showing that the protein conservation within the NHX family is not only restricted to their primary sequence but also extended to their structural flexibility.

#### 3.3.3. Posttranslational Modifications

Posttranslational modifications (PTMs) can adjust localization and protein activity including interactions between proteins and intrinsically disordered regions, hence making plants able to face environmental changes and constraints [28]. These modifications comprise acetylation, phosphorylation, glycosylation, sumoylation, methylation and many other types. The family of VvNHXs harbors multiple sites of activity regulation and PTMs illustrated in Table 6. Glycosylation PTM site is commonly known for biosynthetic processing of transporter proteins in yeast like the NHX1 of* Saccharomyces cerevisiae *which is described as a glycoprotein [29]. In average, two putative N-glycosylation sites were identified in conserved positions of the 6 sequences of VvNHXs; yet supplementary sites of N-glycosylation have been predicted elsewhere for VvNHX1, VvNHX2 and VvNHX3.

Phosphorylation, one of the most common PTMs of proteins [30] has been shown to regulate the activity of animal NHE antiporters [31]. In each member of VvNHXs, putative sites of phosphorylation were found and which ranged from 10 to 18, most of them were of serine type (Table 6; Figure 2(a)); however, only few sites of tyrosine type were detected.

Moreover, various regulatory sites in VvNHX proteins were detected including sumoylation which is a PTM associated with some cellular processes, such as protein stability, transcriptional regulation, response to stress, nuclear-cytosolic transport, and apoptosis.

#### 3.3.4. Tertiary Structure

**Figure 3:**
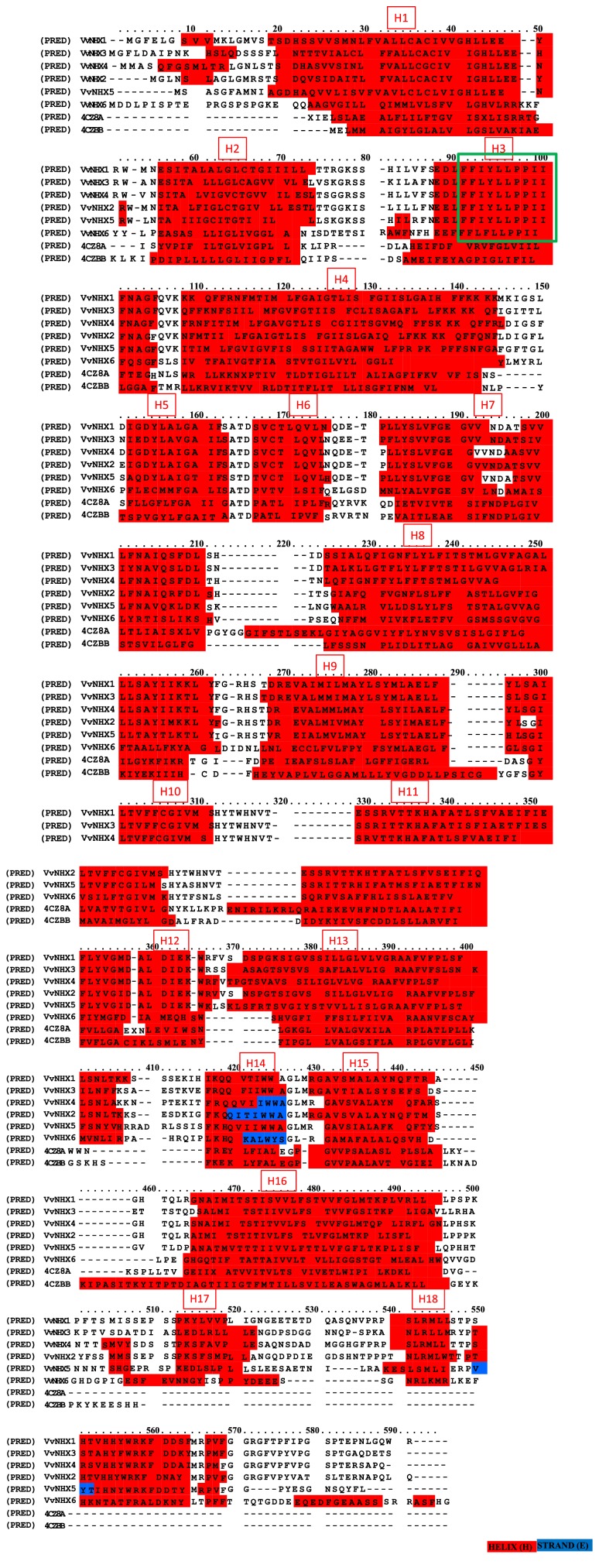
Alignment of the amino acid sequences of the VvNHX family.

Protein Homology/analogY Recognition Engine (PHYRE 2) was used for the prediction of the 3D structure of VvNHX proteins. This tool develops template-based modeling in which a protein is aligned to another of known structure on the basis of patterns of evolutionary variation [19]. Based on homology search, all the VvNHX proteins show homology with the sodium proton antiporter MjNhaP1 from* Methanocaldococcus jannaschii *(c4czbB) with expectation (E) value of ≤ 1 x 10^−10^ (Figure 3, Supplementary file 2). An additional homology with the sodium proton antiporter PaNhaP from* Pyrococcus abyssi* (c4cz8A) was also reported by PHYRE 2. The alignment of VvNHXs with the references sequences of MjNhaP1 (c4czbB) and PaNhaP (c4cz8A) was drawn with PRALINE multiple sequence alignment using DSSP and PSIPRED to perform secondary structure prediction (Figure 3). The selected eight sequences shared similar *α*-helical and extended strands/*β*-sheet content (Figure 3; Table 5). The analysis revealed that the *α*-helices were dominant among the secondary structures (18 helices) except VvNHX2, 4 and 5 which showed only a *β*-sheet structure.

Among the two structural analogs generated by PHYRE 2, c4czbB was chosen to be the appropriate template for homology modeling of the VvNHX antiporter proteins. Wöhlert and collaborators [32] have validated its structure in two different conformations at pH 8 and pH 4 (4CZ9_A and 4CZ8_A), also with bound thallium ion (4CZA_A). Although, no X-ray crystallographic structures for intracellular plant NHX antiporters are available, the intracellular NHXs mediate the H^+^-coupled transport of both Na^+^ and K^+^ [4, 33] while NhaA does not mediate K^+^ transport; meaning that the structure of NHXs must accommodate the relatively larger K^+^ ion [34].

**Figure 4:**
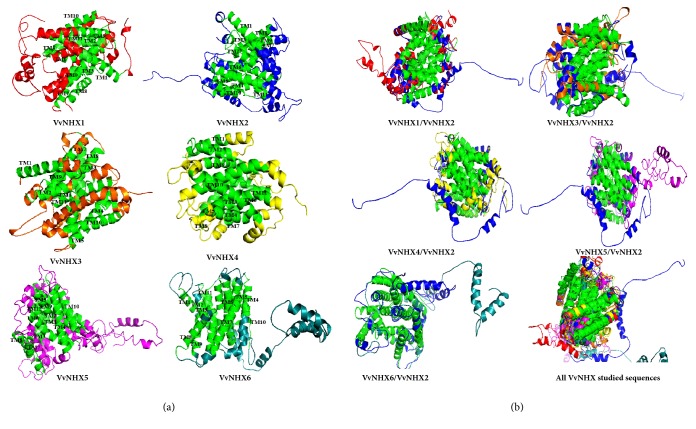
Ribbon representation of the predicted model of* Vitis vinifera* NHX exchangers. (a) *α*-helices and coils are colored from N- to C- terminal by red, blue, orange, yellow, purple, and cyan for, respectively, VvNHX1, VvNHX2, VvNHX3, VvNHX4, VvNHX5, and VvNHX6. The TM domains are colored in green and numbered from 1 to 12. (b) The aligned models are in the order: VvNHX2/1 (blue/ red), VvNHX2/3 (blue/ orange), VvNHX2/4 (blue/ yellow), VvNHX2/5 (blue/ purple), VvNHX2/6 (blue/ cyan), and all VvNHX studied. All TM domains are colored green. Molecular graphics were performed with the PyMOL Molecular Graphics System.

Using the PyMol software, a comparison was made between the template and all VvNHXs (Figure 4). However, the beta strands existing in the* Escherichia coli *NhaA appear to be absent in the VvNHX structure. All the models of VvNHXs consisted mainly of alpha helices with high confidence levels followed by the random coil. As shown in Figure 4(a), the predicted 12 TM domains are localized mainly within alpha helices of VvNHXs except the model of VvNHX3 that harbors only 11 TM domains. Based on the longest sequence in alpha helix, the VvNHX2 protein sequence was selected to align the other sequences of NHX.  Figure 4(b) shows that the TM domains are conserved along all the VvNHX; but C-terminal structures are different from each other remarkably and especially for VvNHX4, 5 and 6.

## 4. Discussion

Our work led to the identification of 6 NHX members in grapevine* Vitis vinifera* L. Based on their subcellular localization, as for Arabidopsis, VvNHXs formed two distinct phylogenetic groups: group I vacuolar (VvNHX1-5) and group II endosomal (VvNHX6).

Like other known intracellular antiporters, the deduced amino acid sequences of VvNHXs family reveal 11-13 putative TM regions, with a consensus of 12 TM.

Though their structures (secondary, tertiary, and quaternary) have yet to be elucidated, the first bacterial crystal structure of* Escherichia coli* Na^+^/H^+^ antiporter NhaA has been established [35, 36]. Recently, Wöhlert et al. [32] have crystallized a sodium/proton antiporter from a single-celled organism* Pyrococcus abyssi* called PaNhaP. They determined the substrate ion in the dimeric PaNhaP at 3.2 Å and have resolved its structure in two different conformations at pH 8 and pH 4. However, to date, no X-ray crystallographic structures for plant NHX antiporters are presented. Nonetheless, two separate topology models have been suggested for AtNHX1. The first model contained nine TM domains with a C-terminal hydrophilic in front of the vacuolar lumen [37] whereas the second topology model suggests that several TM domains of AtNHX1 preserve similar topogenic properties like human NHE1 [38].

Despite the quick progress in molecular identification and biochemical characterization of NHX antiporters, the systematic and the structural analyses of plant NHX antiporter genes have not yet been vigorously investigated. Afore mentioned, the Na^+^/H^+^ antiporter has a vital role in the exchange of Na^+^ for H^+^ across membranes, which is essential for the plant's salt tolerance due to the fact that it keeps cellular ion homeostasis.

Our framework provides new hypotheses and insights into some potential structural features of NHXs from grapevine and offers a basis for further characterizations of the 3D structure of VvNHXs.

Our study pointed out that cation-binding sites and an amiloride-binding site were conserved in all VvNHX proteins. Cation-binding sites shared a high similarity within AtNHX1 homologous. The AtNHX1 has been applied to hold the predicted cation-binding domain as mapped by analogy to other antiporters [39]. Particularly, VvNHX6 displayed the lowest predicted IEP (5.4) which could be linked to its endosomal localization; indeed, according to some authors [40–42], subcellular localization of proteins would be correlated to their IEP values, according to the intracellular pH conditions. In plant cells, there is a decreasing gradient of pH from nuclear to vacuolar compartments, meaning that endosomal compartments (ER, TGN and PVC) are closest to pH 7, matching therefore with the low value of VvNHX6 IEP. Indeed, such neutral pH would be more suitable for VvNHX6 stability and activity; whereas, the acidic vacuolar pH (~ pH 5) is more appropriate to VvNHX1-5 according to their higher IEP. The VvNHX6, like MaNHX6 which belongs to the group II of the plant NHX family [43], does not have the region of CaM-Binding domain and encodes a putative K^+^/H^+^ exchanger. Nevertheless, nor the structural nor the regulatory mechanisms have been unraveled in the plant group II NHX isoforms.

The family of VvNHX contains several sites of activity regulation and PTMs including phosphorylation and glycosylation and shares the same interacting partners. Phosphoproteomic studies of Arabidopsis, barle*y* and rice tonoplast preparations, further suggested the regulation of NHX antiporters by phosphorylation [44]. Knowing that the S471 conserved residue at C-terminus of the rice vacuolar-NHX isoforms was phosphorylated in OsNHX3, this could be confirmed for the grape VvNHX1 and VvNHX2 which have the same type of a conserved residue at serine 462.

Herein, the 3D structure of VvNHXs has been modeled in order to provide insights on their protein architecture and transport function. These antiporters share similarity with crystallized bacterial sodium proton antiporter, despite overall low sequence identity. This hypothesis is based on the observation that cotransporter structures are significantly more conserved in species than linear sequences [45]. However, Sato and Sakaguchi [38] have demonstrated that AtNHX1 has the same topology as human NHE1.

Using a combination of primary structure analysis, secondary structure alignments, and the tertiary structural models, VvNHX revealed conserved 12 TM located within 18 alpha helices. These TM compile a hollow cylinder and embedded in the membrane to provide the antiporter for Na^+^ and H^+^ transport. Recent researches of bacterial NhaA homologs have revealed a lack of *β* sheet, raising interesting questions about the role of the *β* sheet and the importance of the dimerization in these antiporters [46]. This helical phenomenon has been recommended and seen before in other proteins [47] and might be attributed to a particular function. Results support the idea that the core of all VvNHX resembles a sodium proton antiporter. These cation/H^+^ antiporters are essential for diverse functions, such as tolerance to salt stress, K^+^ uptake into vacuoles, pH regulation, and protein targeting [1, 48–51]. Furthermore, it was observed that 3D structures of VvNHX5 (Group I) and VvNHX6 (Group II) were the most divergent especially at their C-terminal structures. In fact, Yamaguchi and coworkers [37] solved the topology of AtNHX1 in which the C-terminus appears to be involved in the determination of the ion selectivity of the transporter. Thus 3D model structures can offer identify core residues of transporters to conclude function.

## Figures and Tables

**Table 1:** NHX family organization in the grapevine genome.

	**VvNHX1**	**VvNHX2**	**VvNHX3**	**VvNHX4**	**VvNHX5**	**VvNHX6**
**Accession number (NCBI)**	AAV36562	NP_001267987	XP_002281198	XP_010659994	CBI26718	XP_002271865

**Length (aa)**	541	538	541	540	524	529

**MW (kD)**	60.1	59.6	59.4	59.5	59.4	58.4

**PI**	7.2	7.7	6.2	8.8	8.6	5.4

**TM regions**	12	12	13	11	12	13

**Subcellular Localization WoLF PSORT**	plas: 5, vacu: 3, E.R.: 3, cyto: 1, mito: 1, golg: 1	plas: 10, vacu: 2, mito: 1, E.R.: 1	vacu: 9, plas: 3, cyto: 1, mito: 1	plas: 9, vacu: 2, E.R.: 2, mito: 1	plas: 4, E.R.: 4, vacu: 3, cyto: 1, mito: 1, golg: 1	Plas:13, cyto: 1

**CELLO Prediction**	Plasma Membrane	Plasma Membrane	Plasma Membrane	Plasma Membrane	Plasma Membrane	Plasma Membrane

**Table 2:** Description of the various protein motifs found by Meme v4.8.2.

**Motif**	**Role**	**Width**	**Best possible match**
1	aspartate-alanine exchange (Asp-Al_Ex)	46	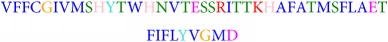

2	Monovalent Cation:Proton Antiporter-1 (CPA1)	36	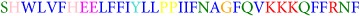

3	-	50	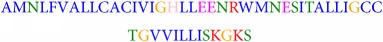

4	Monovalent Cation:Proton Antiporter-1 (CPA1)	27	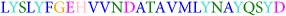

5	-	50	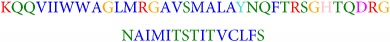

6	-	36	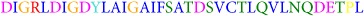

7	-	36	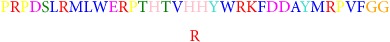

8	-	50	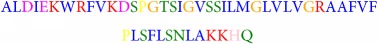

9	-	36	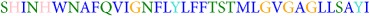

10	-	36	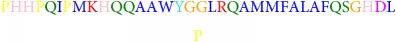

**Table 3:** Amino acid sequence similarity (%) among members of the grapevine NHX family.

	**VvNHX1**	**VvNHX2**	**VvNHX3**	**VvNHX4**	**VvNHX5**	**VvNHX6**
**VvNHX1**	100	81	65	79	61	32

**VvNHX2**	-	100	64	77	60	33

**VvNHX3**	-	-	100	66	59	30

**VvNHX4**	-	-	-	100	61	31

**VvNHX5**	-	-	-	-	100	30

**VvNHX6**	-	-	-	-	-	100

**Table 4:** Amino acid composition of VvNHX proteins.

**Amino acid composition**	**NHX1**	**NHX2**	**NHX3**	**NHX4**	**NHX5**	**NHX6**
Ala (A)	5.7%	5.9%	8.3%	6.9%	6.9%	7.4%

Arg (R)	3.3%	3.0%	3.0%	4.1%	3.6%	3%

Asn (N)	2.6%	3.5%	3.3%	3.3%	3.4%	3.2%

Asp (D)	3.1%	3.0%	4.1%	3.1%	2.7%	3.2%

Cys (C)	0.9%	0.9%	1.3%	1.1%	1.0%	0.8%

Gln (Q)	2.4%	2.8%	2.0%	2.8%	2.5%	2.8%

Glu (E)	3.9%	3.2%	3.5%	2.6%	3.6%	5.3%

Gly (G)	6.7%	7.6%	6.3%	7.2%	6.1%	8.3%

His (H)	2.6%	2.0%	1.7%	2.4%	2.9%	2.6%

Ile (I)	7.9%	8.9%	7.8%	7.2%	8.2%	7.2%

Leu (L)	11.8%	13.0%	13.1%	11.7%	13.4%	11.9%

Lys (K)	3.7%	3.3%	3.9%	2.6%	3.4%	2.6%

Met (M)	3.9%	3.3%	2.4%	3.5%	2.1%	3.4%

Phe (F)	7.9%	8.4%	8.1%	8.5%	7.6%	9.5%

Pro (P)	3.7%	3.9%	2.6%	3.3%	2.7%	4.0%

Ser (S)	10.5%	8.9%	10.2%	10.0%	9.4%	9.1%

Thr (T)	6.7%	7.4%	7.4%	7.2%	8.2%	5.3%

Trp (W)	1.5%	1.3%	1.1%	1.1%	1.5%	0.6%

Tyr (Y)	2.6%	3.0%	3.0%	2.6%	3.4%	3.2%

Val (V)	7.8%	6.5%	7.0%	8.7%	7.4%	6.6%

résidus charge (+) (Asp + Glu)	38	33	41	31	33	45

résidus charge (-) (Arg + Lys)	38	34	37	36	37	30

Δ charge (pH 7)	0	-	4+	5-	4-	15+

**Table 5:** Details of secondary structure of VvNHX proteins according to the NPS secondary structure prediction method (GORIV).

**Protein**	**α** **-helix**	**Extended strand**	**Random coil**
**VvNHX1**	32,10	23,05	44,80

**VvNHX2**	33,27	28,81	28,44

**VvNHX3**	41,77	15,34	42,88

**VvNHX4**	27,22	30,56	42,22

**VvNHX5**	30,34	25,76	43,89

**VvNHX6**	38,75	18,15	43,10

**Table 6:** Putative sites for posttranslational modifications in VvNHXs.

**Protein**		**N-acetylation**	**N-glycosylation**	**Predicted Phosphorylated Sites**			**Sumoylation**
				Serine (S)	Threonine (T)	Tyrosine (Y)	
**VvNHX1**	Sites	-	3	13	5	0	5
	Positions	-	50, 293, 368				19, 149, 522, 462, 113

**VvNHX2**	Sites	-	3	9	8	1	6
	Positions	-	47, 290, 365				380, 385, 144, 473, 519, 459

**VvNHX3**	Sites	-	4	10	2	0	4
	Positions	-	21, 52, 111, 295				111, 141, 515, 107

**VvNHX4**	Sites	-	3	5	7	0	4
	Positions	-	15, 51, 294				515, 469, 376, 106

**VvNHX5**	Sites	1	2	7	3	2	3
	Positions	2	44, 287				98, 195, 46,

**VvNHX6**	Sites	-	2	6	1	3	3
	Positions	-	71, 300				48, 110, 487

## Data Availability

The accession numbers of VvNHX sequences data used to support the findings of this study are included within the article.

## Abbreviations

CPA1: Cation: Proton Antiporter-1
MEME: Multiple Expectation Maximization for Motif Elicitation
TM: Transmembrane
PTM: Posttranslational Modification.

## Supplementary Materials

Supplementary Materials Supplementary File 1: alignment global of VvNHX proteins (.png). Supplementary File 2: details of tertiary structure of VvNHXs proteins (.doc).
