# Supplementary material for: Computational Approach for Structural Feature Determination of Grapevine NHX Antiporters
Source: Biomed Res Int. 2019 Jan 9;2019:1031839. doi: 10.1155/2019/1031839 (PMC6343165; doi:10.1155/2019/1031839)
Supplement: Supplementary Materials — Supplementary File 1: alignment global of VvNHX proteins (.png). Supplementary File 2: details of tertiary structure of VvNHXs proteins (.doc). [file 1031839.f1.doc]

Supplementary file2 :


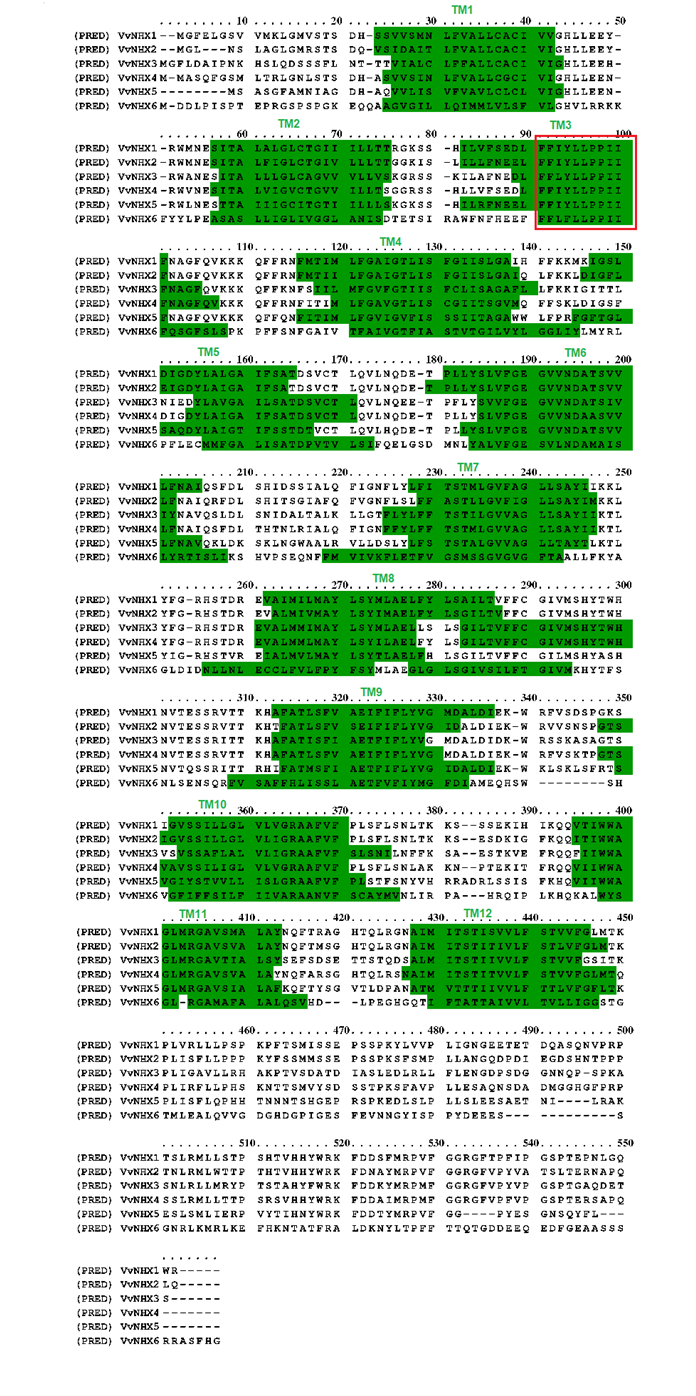


Supplementary file2 :

|  | Description | Confidence (Residues aligned) | Query cover (%identity) | Template |
| --- | --- | --- | --- | --- |
| VvNHX1 | Structure of the sodium proton antiporter MjNhaP1 from *Methanocaldococcus jannaschii* at pH 8 | 100% (22 🡪 452) | 79% (18%) | c4czbB_ |
|  | Structure of the sodium proton antiporter PaNhaP from *Pyrococcus abyssii* at pH 8 | 100% (24 🡪 447) | 78% (23%) | [c4cz8A_](http://www.sbg.bio.ic.ac.uk/phyre2/html/flibview.cgi?pdb=c4cz8A_) |
| VvNHX2 | Structure of the sodium proton antiporter MjNhaP1 from *Methanocaldococcus jannaschii* at pH 8 | 100% (19 🡪 449) | 79% (18%) | c4czbB_ |
|  | Structure of the sodium proton antiporter PaNhaP from *Pyrococcus abyssii* at pH 8 | 100% (24 🡪 444) | 78% (23%) | [c4cz8A_](http://www.sbg.bio.ic.ac.uk/phyre2/html/flibview.cgi?pdb=c4cz8A_) |
| VvNHX3 | Structure of the sodium proton antiporter MjNhaP1 from *Methanocaldococcus jannaschii* at pH 8 | 100% (23 🡪 448) | 79% (17%) | c4czbB_ |
|  | Structure of the sodium proton antiporter PaNhaP from *Pyrococcus abyssii* at pH 8 | 100% (27 🡪 443) | 78% (23%) | [c4cz8A_](http://www.sbg.bio.ic.ac.uk/phyre2/html/flibview.cgi?pdb=c4cz8A_) |
| VvNHX4 | Structure of the sodium proton antiporter MjNhaP1 from *Methanocaldococcus jannaschii* at pH 8 | 100% (23 🡪 443) | 79% (16%) | c4czbB_ |
|  | Structure of the sodium proton antiporter PaNhaP from *Pyrococcus abyssii* at pH 8 | 100% (25 🡪 448) | 78% (22%) | [c4cz8A_](http://www.sbg.bio.ic.ac.uk/phyre2/html/flibview.cgi?pdb=c4cz8A_) |
| VvNHX5 | Structure of the sodium proton antiporter MjNhaP1 from *Methanocaldococcus jannaschii* at ph 8. | 100% (16 🡪 448) | 79% (16%) | c4czbB_ |
|  | Structure of the sodium proton antiporter PaNhaP from *Pyrococcus abyssii* at pH 8 | 100%(20🡪 444) | 78% (22%) | [c4cz8A_](http://www.sbg.bio.ic.ac.uk/phyre2/html/flibview.cgi?pdb=c4cz8A_) |
| VvNHX6 | Structure of the sodium proton antiporter MjNhaP1 from *Methanocaldococcus jannaschii* at pH 8 | 100% (23 🡪 447) | 79% (19%) | c4czbB_ |
|  | Structure of the sodium proton antiporter PaNhaP from *Pyrococcus abyssii* at pH 8 | 100% (28 🡪 442) | 78% (20%) | [c4cz8A_](http://www.sbg.bio.ic.ac.uk/phyre2/html/flibview.cgi?pdb=c4cz8A_) |
